# Recent advances in the characterization of plant transcriptomes in response to drought, salinity, heat, and cold stress

**DOI:** 10.12688/f1000research.18424.1

**Published:** 2019-05-14

**Authors:** Khurram Bashir, Akihiro Matsui, Sultana Rasheed, Motoaki Seki

**Affiliations:** 1 Plant Genomic Network Research Team, CSRS, RIKEN, Yokohama, Tsurumi-ku, Kanagawa, 230-0045, Japan; 2 Plant Epigenome Regulation Laboratory, CPR, RIKEN, Wako, Saitama, 351-0198, Japan; 3 Kihara Institute for Biological Research, Yokohama City University, Totsuka-ku, Yokohama, 244-0813, Japan; 4 Core Research for Evolutionary Science and Technology, Japan Science and Technology Agency, Kawaguchi, Saitama, 332-0012, Japan

**Keywords:** Transcriptomic analysis, Drought Stress, Heat Stress, Cold Stress, Salinity Stress, Microarray Analysis, RNA Seq Analysis

## Abstract

Despite recent advancements in plant molecular biology and biotechnology, providing food security for an increasing world population remains a challenge. Drought (water scarcity), salinity, heat, and cold stress are considered major limiting factors that affect crop production both qualitatively and quantitatively. Therefore, the development of cost-effective and environmentally friendly strategies will be needed to resolve these agricultural problems. This will require a comprehensive understanding of transcriptomic alterations that occur in plants in response to varying levels of environmental stresses, singly and in combination. Here, we briefly discuss the current status and future challenges in plant research related to understanding transcriptional changes that occur in response to drought, salinity, heat, and cold stress.

## Introduction

Adverse environmental conditions such as unfavorable temperatures (high or low), drought stress/water shortage, and salt stress negatively affect agricultural production and reduce crop yield, both qualitatively and quantitatively. Increases in temperature and water scarcity are predicted to occur as an outcome of climate change and thus will pose a serious challenge to agricultural production worldwide. Understanding the molecular response of plants to abiotic stresses and using this knowledge to develop crop plants that can adapt to and maintain high yields under these adverse environmental conditions has always been a major objective for molecular breeders ^1– 7^. Despite concerted efforts and significant discoveries, the challenge still persists, highlighting its complexity and the need to develop novel approaches to ameliorate the damage resulting from environmental stress. Some plant species have the ability to successfully survive in challenging environments by evolving complex and integrated cellular or physiological processes that are controlled by regulatory and functional genes. It may be difficult, however, for plants to respond to the rapid changes occurring because of climate change as evolution is a gradual process.

Despite a comprehensive knowledge of the mechanisms governing cellular responses to abiotic stresses, our understanding of stress signal perception during the early stages of abiotic stress response is relatively poor ^8^. Transcriptomic analyses have provided an in-depth knowledge of the cellular and molecular responses underlying plant adaptation to environmental stresses ^9– 12^.

## Transcriptomic changes under drought, salinity, cold, and heat stress

Abiotic stresses trigger significant molecular and physiological changes in plants, including quick transcriptomic and metabolic adjustments, adjustments in osmotic potential, reduction of leaf turgor pressure, and ultimately the slowing down or cessation of plant growth ^13, 14^. The reduction in growth may affect some tissues more severely than others. For example, while shoot growth is severely inhibited in response to drought stress, roots may continue to grow in an effort to increase water absorption ^15, 16^. Roots of soil-grown *Arabidopsis* undergo a greater diversity of transcriptomic changes compared with shoots in response to drought stress conditions, revealing several novel candidate genes that might regulate root response to drought stress ^11, 17^. These studies indicate that plant tissues that initially sense changes in environmental conditions may undergo a greater number of transcriptomic changes relative to tissues that subsequently sense or detect the stress at a later stage. For example, early stages of drought and salt stress principally affect roots whereas heat and cold stress may initially be sensed by shoots.

The expression of genes belonging to diverse functional and regulatory groups, such as transcription factors, protein kinases, and phosphatases ^1, 9, 11, 18– 21^, are altered in response to abiotic stress conditions. Changes in the expression of genes encoding enzymes regulating osmolytes, late embryogenesis abundant (LEA) proteins, aquaporins, and reactive oxygen species (ROS) scavengers and chaperones that protect the integrity of cell membranes and ensure the maintenance of ion transport and balances are also variably observed in response to different abiotic stresses. Moreover, small peptides, plant hormones, and non-coding RNAs that regulate gene expression and signal transduction act as major players and key components in the mechanisms underlying abiotic stress response ^9, 22– 24^. Plant hormones, such as abscisic acid (ABA), play an integral role in plant response to various types of abiotic stresses, including salt stress, drought stress, and extreme temperatures. A common factor among these stresses is that they all induce osmotic stress in plant cells ^25^. Intracellular sensing and signal transduction of ABA result in the activation of downstream effectors, including transcription factors and ion channels, which implement important adaptive responses, such as stomatal closure, osmoprotectant synthesis, and the induction of a broad range of stress-responsive genes, that allow plants to withstand reduced water availability ^25^. ABA biosynthesis and its transport and accumulation all increase in plant tissues in response to water deficit/water deficiency/dehydration conditions and other abiotic stresses, providing the ability of plants to adapt to the stress by regulating the internal water status in plants ^26^. ABA-independent responses to various abiotic stresses are also crucial and are regulated mainly by dehydration-responsive element/C-repeat (DRE/CRT) and DRE-/CRT-binding protein 2 (DREB2) transcription factors ^27^. Recently, the role of peptide hormones and small open reading frames (sORFs) in regulating the plant response to different abiotic stresses, such as drought and salt stress, has been reported ^12, 23, 24, 28^. The role of non-coding RNAs has also been extensively examined in recent years ^29^. These RNAs play a functional role in diverse plant responses, such as the regulation of transcription, splicing and nuclear structure, and epigenomics. Thus, care should be taken to choose a technology platform that provides the ability to monitor changes in sORFs, non-coding RNAs, and so on when designing experiments to study plant response to environmental stress.

Alternative splicing may allow the synthesis of more than one kind of protein from the same gene when splice sites are differentially recognized and more than one transcript, and potentially multiple proteins, are generated from the same pre-mRNA ^25^. Different RNA sequence elements are associated with the potential for alternate splicing; however, changes in chromatin structure, histone modifications, and regulation of transcription rates also play important roles in alternate splicing ^18, 25, 30– 32^. The alteration of histone modifications and DNA methylation in plants that is associated with adaptation to environmental changes is coordinated with changes in the expression of stress-responsive genes. Several chromatin modifications in plants, such as acetylation, methylation, phosphorylation, and SUMOylation, occur in response to drought, salinity, and both high and low temperature ^18^. Thus, a comprehensive understanding of these changes would increase the ability to develop precise strategies for fine tuning the genome to increase stress tolerance in crop plants.

## Factors to consider while generating and using transcriptomic data

The above discussion provides a brief overview of transcriptomic alterations affecting abiotic stress tolerance and adaptability in plants in response to different environmental cues. Importantly, however, there is the question of how the available transcriptomic data and the discovery of new factors and elements can be used to produce crop plants that are more resilient to abiotic stress. Besides generating additional unique datasets, the current major challenge is how the existing data can be used for crop improvement. The major approach to obtaining transcriptomic information over the past two decades has been through microarray analysis, which still represents a suitable approach based on the ease of data analysis, information handling, and cost-effectiveness. Tilling or customized arrays (or both) allow one to delve deeper in the plant genome, providing information on non-coding RNAs, sORFs potentially coding small peptides, and other transcriptional elements in different crops ^9, 33, 34^. RNA sequencing (RNA-seq) analysis, however, is gradually becoming the method of choice because of its ever-increasing cost-effectiveness, availability of more in-depth data analysis tools, its suitability for focusing on alternate splicing, and its ability to examine the whole genome. Thus, RNA-seq analysis, which provides precise information on the transcriptomic changes that occur in response to abiotic stress, is now the method of choice for the majority of plant scientists ^35– 38^.

In addition to the technical aspects associated with transcriptomic studies, other factors should be considered in order to advance our understanding of abiotic stress tolerance mechanisms in plants. Transcriptomic data can be used to identify novel genes and promoters ^11, 39, 40^. In this regard, genes whose function has not yet been determined could be comprehensively studied to reveal their functional roles under different stress conditions. These data could also be used to identify genetic markers that would advance the tools available for marker-assisted breeding. Such knowledge could also be used to identify cost-effective and environmentally safe chemicals, such as acetic acid and ethanol, that enhance drought and salt stress tolerance ^39, 41^.

Plants may respond differently to the same abiotic stress at different developmental stages in their life cycle. For example, plant response to abiotic stress may be significantly different during their vegetative versus reproductive stages of growth. The severity of the applied stress should also be taken into account when analyzing or using generated data. Moderate and long-term drought stress may only partially trigger specific genes compared with a severe and rapid drought stress. Even data collected at different time points during the same stress can be significantly different ^11^ as transcriptional changes in response to abiotic stress are also regulated by diurnal rhythms ^42, 43^. An *Arabidopsis* circadian oscillator is regulated by light, temperature, changes in metabolite concentrations such as sugars, hormones such as ethylene, and ions such as Ca ^2+^ ^44^. As these factors change in response to the abiotic stresses discussed above, the expression of circadian oscillator–related genes is also subject to change in response to these stresses. The combined effect of temperature and day length shapes the dynamics of the *Arabidopsis halleri* transcriptome and adaptation to seasonal changes in a natural habitat ^45^ and it seems reasonable to predict that crop plants would respond in a similar manner.

Recent advances indicate that not only different tissues but different cells within the same tissue may exhibit a different transcriptomic profile, highlighting the necessity of single-cell transcriptomic analyses ^46^. This variation between cells may be due to the phenomenon that genes are not continuously transcribed but rather undergo short intervals of “on” and “off” states, thus making transcriptional monitoring difficult ^46^. Single-cell transcriptomic data analysis, such as evaluating the unsupervised clustering of single-cell RNA-seq data which is used to identify putative cell types, is also particularly challenging ^47^. Individual cells in *Arabidopsis* roots and embryos have been profiled, providing a comprehensive overview of the transcriptome and revealing the diversity in gene expression among cell types across different developmental stages ^48, 49^. These examples indicate that, despite technical difficulties, single-cell analysis could enhance the reliability of transcriptomic comparisons among different plant species by addressing the variation present in tissues or organs (or both) ^50^. In the future, a greater effort will be needed to uncover differences in gene expression among tissues and cells, particularly in response to abiotic stress.

## Future prospects

**Figure 1.**
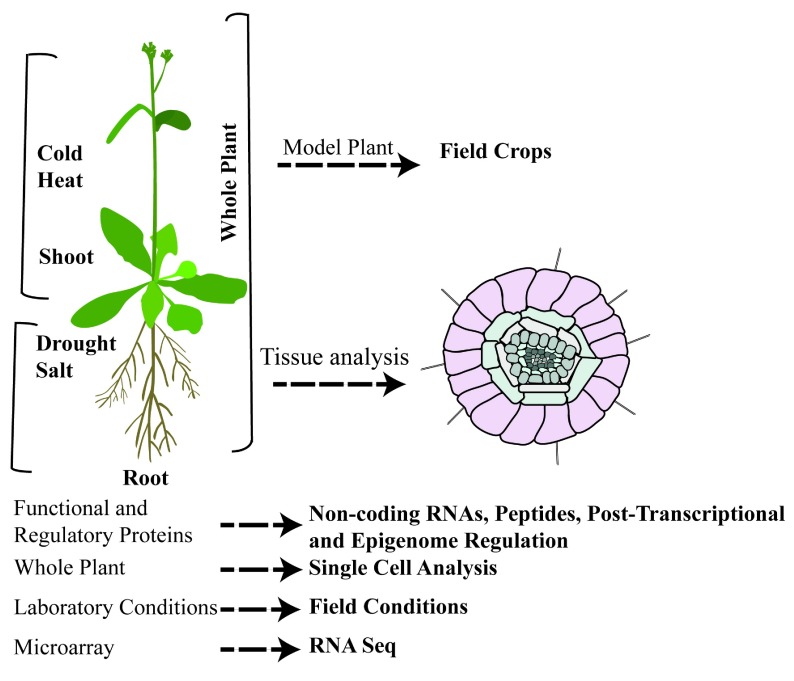
Transcriptomic analysis under drought, salinity, heat, and cold stress. The focus of research is shifting from model plants to field crops; from whole plants to tissue and single-cell analysis; from functional and regulatory proteins to non-coding RNAs, small peptides, and post-transcriptional and epigenomic regulation; from laboratory conditions to field conditions; and from microarray to RNA sequencing (RNA-seq) analysis.

A significant amount of transcriptomic data obtained from model plants is already available, so there is a distinct need to focus on crop plants ( Figure 1). Several groups have used RNA-seq analysis in recent years to investigate transcriptional changes in different crop plants and a few examples will now be discussed. Comparative transcriptome analysis of several chickpea genotypes at different developmental stages identified the upregulation of 4954 genes in drought-tolerant and 5545 genes in salinity-tolerant genotypes ^51^. A similar study used an RNA-seq approach to identify differentially expressed genes in cold-tolerant and cold-sensitive varieties of sorghum in response to cold stress and control conditions ^52^. Abiotic stresses altered the transcriptome of poplar through alternative splicing, differential intron retention, and isoform ratio switching in a stress- or tissue-specific manner (or both) ^53^. RNA-seq analysis in rice revealed the upregulation of genes encoding heat shock proteins and heat shock factors during anthesis and the downregulation of genes, such as transcription factors, or genes related to signal transduction and metabolic pathways in a heat-tolerant rice cultivar compared with a heat-sensitive cultivar ^54^. Genes potentially related to heat stress tolerance in spinach have also been identified ^55^. Several studies focused on wheat and triticale transcriptional or metabolic changes (or both) to elucidate the stress response mechanisms ^56– 58^. Transcriptome profiling has also revealed temporal changes in the expression of genes that contribute to heat and drought acclimation in wheat ( *Triticum aestivum* L.) ^59^. Comparison of the genomes of different pearl millet varieties could serve as a resource for the improvement of stress tolerance in arid environments ^60^. The creation and maintenance of databases focusing on a particular group of genes have also been reported. A rice kinase database (RKD) provides the ability to access metadata obtained from National Center for Biotechnology Information Gene Expression Omnibus (NCBI GEO) expression datasets, thus facilitating the in-depth transcriptomic analysis of kinase-encoding genes in diverse rice tissues and in response to biotic and abiotic stresses and hormone treatments ^61^. Transcriptional databases covering various developmental stages and the response to various stresses have also been maintained for crops such as rice ^62^, wheat, barley, maize, potato, tomato, grapes, peanut, strawberry, and poplar ^63, 64^. Importantly, transcriptomic information obtained from plants grown on Murashige and Skoog (MS) medium and hydroponic solutions and in soil in response to different stress conditions is significantly different ^62^. Several groups have monitored transcriptomic changes in crop plants under field conditions ^62, 65^; however, a greater effort is needed to obtain data on plant response to abiotic stress factors under field conditions at different developmental stages to obtain broad-spectrum applicability of the observed changes in gene expression that could be used to produce crops that are more resilient to abiotic stress. This approach would help to identify and regulate genes that have the ability to impact stress tolerance under diverse environmental conditions. Moreover, confirmation of transcriptomic changes by other -omic technologies, such as metabolomics ^66^ and proteomics ^67^, could significantly improve the reliability and applicability of transcriptomic data, thus leading to the development of sustainable solutions. The utilization of such information would greatly facilitate the ability to address the challenges posed by climate change and speed up the efforts to breed crop plants that can maintain high yields under limiting growing conditions.
